# Effects of Heliox in Stable COPD Patients at Rest and during Exercise

**DOI:** 10.1155/2012/593985

**Published:** 2012-10-10

**Authors:** Matteo Pecchiari

**Affiliations:** Dipartimento di Fisiopatologia e dei Trapianti, Università degli Studi di Milano, Via Mangiagalli 32, 20133 Milano, Italy

## Abstract

Heliox has been administered to stable chronic obstructive pulmonary disease (COPD) patients at rest and during exercise on the assumption that this low density mixture would have reduced work of breathing, dynamic hyperinflation, and, consequently, dyspnea sensation. Contrary to these expectations, beneficial effects of heliox in these patients at rest have been reported only sporadically, and the majority of the studies performed until now suggests that heliox is not a therapeutic option in spontaneously breathing resting COPD patients. On the other hand, when it is administered to COPD patients exercising at a constant work rate, heliox systematically decreases dyspnea sensation, and, often but not always, increases exercise tolerance. For these reasons, heliox has been evaluated as a non pharmacological tool to power rehabilitation programs. The conflicting results provided by the published trials probably point at a substantial heterogeneity of the COPD patients population in terms of respiratory mechanics and gas exchange. Therefore, further studies, aimed to the identification of mechanisms conditioning the response of exercising COPD patients to heliox, are warranted, before heliox administration, which is costly and cumbersome, can be routinely used in rehabilitation programs.

## 1. Introduction

The clinical use of helium-oxygen mixtures (heliox) in patients with asthma or with larynx or trachea obstruction was first described in 1934 by Barach [1]. From then, the interest in the clinical use of heliox declined, in part because of the discovery of bronchodilators and in part because of the loss of many locations of natural helium during the Second World War [2]. The enthusiasm for heliox resurged in the late 1980s, concomitantly with increased mortality from asthma [3]. At present, the use of heliox has been advocated for a number of conditions, like upper airway obstruction, croup, acute asthma, and postextubation stridor [4]. Heliox has been administered in chronic obstructive pulmonary disease (COPD) patients on the assumption that this gas mixture, because of its low density, is able to reduce pulmonary resistance. It is thus worth to briefly revise the physical properties of heliox and their impact on the dynamics of the respiratory system.

## 2. Physical Properties of Helium

 Helium is the lighter element after hydrogen and it heads the noble gas series in the periodic table, with an atomic number of 2 and an atomic weight of 4 g mol^−1^. Due to its low melting and boiling point, at ambient temperature and pressure it exists as a gas. Helium is considerably less dense and slightly more viscous than air: dry, at 37°C and 1 atm, its density is 0.157 Kg m^−3^ and its viscosity 204 *μ*P (for comparison, in the same conditions air density and viscosity are 1.134 Kg m^−3^ and 190 *μ*P, resp.). The solubility coefficient of helium in water is very low compared to nitrogen, oxygen, and carbon dioxide (at 37°C 0.0014, 0.014, 0.03 and 1 g/L, resp.).

 Because of its low solubility, helium passes the alveolar-capillary membrane very slowly, despite its diffusibility is greater than that of oxygen, carbon dioxide, and even nitrogen. The single orbital of helium is completely filled by two electrons, so helium does not form compounds. It is regarded as metabolically inert and appears as a colourless, odourless, and tasteless gas. Unlike xenon, it is devoid of anesthetic properties. As a therapeutic gas, helium is used to replace nitrogen as a carrier gas for oxygen. The percentage of oxygen in heliox (which will be indicated from now on as subscript) should be at least 20% to prevent hypoxia, and no more than 40%, because beyond this value heliox is not likely to exert any relevant clinical effect [5]. The density of a helium-oxygen mixture can be obtained as the weighted mean of oxygen and helium densities [6]. At 37°C and 760 mmHg, 20%, 30%, and 40% O_2_ in He have densities of 0.377, 0.488, and 0.600 Kg m^−3^, respectively. In contrast, the viscosity of heliox mixtures cannot be obtained as the weighted mean of oxygen and helium viscosities, because the viscosity of a gas mixture is higher than the average viscosity of individual gases. By using the semiempirical formula of Wilke [7], at 37°C and 760 mmHg, the viscosities of 20%, 30%, and 40% O_2_ in He result 225, 226, and 226 *μ*P, respectively.

 From a technical point of view, it is worth to note that because of the different viscosity of air and heliox, Fleish type flowmeters should be calibrated with each gas mixture. At 20°C, the ratio between the viscosities of water vapour saturated heliox_21_ and air is ~1.15, so, if the flowmeter is not recalibrated before use with heliox_21_, flow will be overestimated by ~15%. Moreover, a considerable error can be introduced if the flowmeter is calibrated using dry heliox_21_ without previous humidification, because the viscosity of the dry mixture is greater than that of the water saturated mixture. In this case, expiratory flow will be underestimated by ~8% [8].

## 3. Fluid Dynamics

 Consider a tube of diameter *D* in which a fluid of density *ρ* and viscosity *η* flow steadily. The regime of the flow inside the tube (laminar, transitional, or turbulent) depends on a dimensionless quantity called Reynolds number (Re), which is the ratio of inertial to viscous forces as

(1) Re=ρDvη=4πρV˙ηD,

where *v* is the velocity and $\dot{V}$ is the flow. Indicatively, flow is laminar if Re is less than ~2300, overtly turbulent if Re is more than ~4000, and transitional if Re is between ~2300 and ~4000. The pressure difference required to generate a given flow is greater if the flow regime is turbulent than if it is laminar, because in the former condition (a) the boundary layer is thinner and the shear near the wall is increased, and (b) the fluid elements experience accelerations which are dissipated as heat. Using the description of the conductive airways provided by Weibel [9] to calculate Re for each generation at a given flow, it can be predicted that, at rest, transitional or turbulent flow is confined to the trachea, because only in this location Re is greater than 2300. When ventilation is increased as during exercise, Re increases in each generation and turbulence extends distally in the central airways.

 Predicting the flow regime in the tracheobronchial tree on the basis of the Reynolds number actually overestimates the amount of laminar flow present, because the establishment of Poiseuille flow is not immediate at the entrance of each airway generation. Assuming a flat velocity profile of airflow at the entrance of an airway, a fully established laminar flow can be found only after a certain length from the entrance (*L*_*e*_) given by

(2) Le=k2ρDvηD=k2ReD,

where *k*_2_ is a constant depending on Re. For Re less than 2300 and greater than 50, *k*_2_ is ~0.03; for Re less than 50, the ratio *L*_*e*_/*D* is constant and ~1.5. As *L*_*e*_ so calculated is greater than the anatomical length of the large conducting airways, the part of the tracheobronchial tree in which flow is turbulent or transitional should be substantially greater than that estimated solely by the computed Re.

 Because the kinematic viscosity of heliox_21_ is ~4 times that of air, substitution of air with heliox_21_ causes a 4 times decrease of Re in each airway generation, possibly causing the transition of turbulent to laminar flow at some locations. Moreover, by reducing *L*_*e*_, heliox can further reduce the extension of the part of the tracheobronchial tree involved by turbulence.

 The ability of heliox to keep the airflow laminar is not the sole reason of its favourable effects on respiratory mechanics. Actually, heliox is able to decrease airway resistance even if the flow regime remains turbulent.

 Independently of the flow regime, the relation between the flow ($\dot{V}$) and the pressure difference between the inlet and the outlet of a circular tube (Δ*P*) is given by

(3) $$\begin{array}{c} \dot{V}=\pi \sqrt{\frac{1}{8}\frac{\Delta P}{L}\frac{D^{5}}{\rho f}}, \end{array}$$

where *f* is the friction factor [6]. The relation between *f* and Re is graphically represented in the Moody's diagram.

 For fully established laminar flows, *f* = 64/*Re*. In this case (3) becomes the well-known Poiseuille's equation as follows

(4) $$\begin{array}{c} \dot{\text{V}}=\frac{\pi }{128\text{L}\eta }\Delta \text{P}{\text{D}}^{4}. \end{array}$$

In the region of transition between laminar and turbulent flow, *f* depends on both wall roughness and Re. For a fully established turbulent flow, if the wall of the tube is rough, *f* is independent on the Reynolds number and dependent only on wall roughness; if the wall is smooth, *f* is proportional to Re^-1/4^.

 As *ρ* appears in the denominator of (3) and *f* is proportional to *ρ* ^−1^ only when laminar flow is present, the replacement of air with heliox should decrease the pressure difference necessary to generate a given flow, even if the flow regime remains overtly turbulent. Therefore, if the flow is transitional or overtly turbulent, density dependence is always present at some variable degree, according to the flow regime and to the characteristics of the airways. If the airflow is purely laminar, no benefit of heliox should be expected; conversely, airway resistance should increase, because of the increased viscosity of heliox relative to air.

## 4. Density-Dependence of Maximal Expiratory Flow in Normal Subjects and COPD Patients

 Beside reducing the pressure difference between the alveoli and the mouth which should be developed in order to generate a given flow and, consequently, the metabolic cost of breathing, heliox is potentially able to increase the maximal ventilation available to a subject. This effect is highly desirable in COPD subject, in whom ventilation can be a constraint of physical performance. In contrast, most normal subjects do not use the maximal flows available even at peak exercise [10].

 The density-dependence of the maximal flows can occur only if certain conditions are met. During forced expirations, dynamic compression of the intrathoracic airways takes place, and flows become effort-independent when the pulmonary volume is less than 80% of the vital capacity. In this volume range, expiratory flow limitation occurs.

 Flow limitation may result from two mechanisms: (a) the coupling between airways compliance and convective acceleration of gas (wave-speed theory) [11], or (b) the coupling between airways compliance and viscous pressure losses [12]. In case (a), the maximal flows are inversely proportional to the square root of the gas density, as the wave-speed theory states that the maximal flow (${\dot{V}}_{\max}$) inside a compliant tube is that at which the local velocity of the fluid is equal to the propagation velocity of a small disturbance travelling on the wall of the tube, according to the following equation:

(5) $$\begin{array}{c} {\dot{V}}_{\max}=A\sqrt{\frac{A}{\rho }\frac{dP_{\text{tm}}}{dA}}, \end{array}$$

where *A*(*dP*_tm_/*dA*) is the elastic module of the tube and *A* the cross section.

 Conversely, in case (b) the maximal flows are density-independent, as the viscous pressure losses are determined solely by the viscosity and by the geometrical characteristics of the airways, as long as the flow-regime remains laminar.

 When a normal subject forcedly expires, as long as lung volume stays in the upper two-thirds of his vital capacity, the choke point, that is, the part of the airways where dynamic compression actually limits expiratory flow, is found in the central airways, where the cross-sectional area is small, and the lateral pressure drop is largely due to convective acceleration. In this volume range, flow limitation is due to the wave-speed mechanism, and if air is replaced by heliox, maximal flows increase. In the lower third of the vital capacity, the choke point moves upstream in the peripheral airways, where the cross-sectional area is large, the flow is laminar, and the viscous mechanism is predominate. In this case the maximal flows become density-independent.

 During the evolution of the disease, COPD patients experience a progressive reduction of their maximal expiratory flows that may become so low that flow-limitation is present even at rest. It is believed that the disease first arises in the peripheral airways, which are the major site of increased resistance in many COPD patients [13–15]. In line with this assumption, when air is replaced by heliox_21_ the increase of maximal expiratory flow at 50% of VC (${\dot{V}}_{\text{max,50\%VC}}$) is generally lower in smokers than in nonsmokers [16]. However, contrary to the expectations, in COPD patients a reduced density-dependence is not a rule. In a sample of 22 COPD patients, density-dependence, defined as an increase of ${\dot{V}}_{\text{max,50\%VC}}$ greater than 20% when air is replaced by heliox_21_, was present in 11 patients [17]. In this study, patients with decreased density-dependence differed from those with normal density-dependence because of smaller vital capacity, large ratio of residual volume to total lung capacity, higher resistance, and lower static lung recoil at total lung capacity. These results suggest that different patterns of airways lesions are present in the COPD population. Even if the disease starts peripherally, central airways can be affected with variable degree, so that their mechanical properties change in a way that during maximal expiration the choke point moves in some patients to the peripheral airways, and in some others remains in the central airways.

## 5. Heliox Breathing at Rest in COPD Patients

 In healthy human subjects at rest, the end-expiratory volume corresponds to the relaxation volume of the respiratory system. In COPD patients, pulmonary hyperinflation, that is, an increase of functional residual capacity above the predicted normal value, is often present, because of reduced lung recoil, as in emphysema, and/or because of dynamic hyperinflation. The latter occurs when the duration of expiration is not sufficient to allow the respiratory system to deflate to its relaxation volume prior to the next expiration, possibly because the time-constant of the respiratory system has increased (increased airway resistance) or the respiratory rate is too high. In COPD patients, dynamic hyperinflation is mainly due to the presence of tidal expiratory flow-limitation, that is, the inability to increase expiratory flow by further increasing the transpulmonary pressure during tidal breathing. The assessment of changes of dynamic hyperinflation is usually made by measuring the opposite changes of inspiratory capacity [18]. Dynamic hyperinflation and concomitant intrinsic positive end-expiratory pressure increase inspiratory work, impair inspiratory muscles function, and adversely affect hemodynamics [19]. All these factors, together with dynamic airways compression, may contribute to dyspnea [20, 21]. 

 Currently, dynamic hyperinflation can be decreased by bronchodilators [22] or, in hypoxemic patients, by oxygen administration, which reduces ventilation. Heliox, by decreasing airway resistance and increasing maximal expiratory flows, could provide further relief to COPD patients.

 Unfortunately, in resting COPD patients, airway resistance during heliox_21_ breathing can decrease [23], or remain substantially unchanged [24]. In contrast, heliox_21_ has been regularly found to decrease airways resistance in healthy subjects at rest [25, 26], in line with the notion that, in a normal respiratory system, the resistance of the central airways, where airflow is transitional or turbulent, accounts for a substantial part of total airway resistance [27].

 Conflicting results have been obtained also regarding the effects of heliox_21_ administration on dynamic hyperinflation in COPD patients at rest. Grapè et al. [23] reported no effect of heliox_21_ on dynamic hyperinflation; conversely, a significant fall of end-expiratory lung volume was detected by Swidwa et al. in 15 patients [28]. It should be noted that some of these patients were studied after hospital discharge for bronchitic exacerbations or coronary artery disease, and most had a forced expired volume in one second (FEV_1_) response to the bronchodilator greater than 20%, an unusual finding in COPD patients. Afterwards, a lack of effect of heliox_21_ on dynamic hyperinflation in COPD patients at rest has been repeatedly reported [29–34]. Recently, one study by Chiappa et al. [35] documented an average 17% increase of inspiratory capacity at rest when air was replaced by heliox_21_. Their 12 COPD patients showed a marked density-dependence of maximal expiratory flows, as heliox_21_ increased peak expiratory flow by 31% and forced expiratory flow between 25 and 75% of the forced vital capacity by 46%.

 The effects of heliox_21_ on tidal expiratory flow-limitation and dynamic hyperinflation have been assessed by Pecchiari et al. and compared to those of a bronchodilator in 22 stable COPD patients at rest [29]. In all the patients who were flow-limited, heliox_21_ did not decrease dynamic hyperinflation, independent of posture. In 9 out of 13 patients who were flow-limited in the sitting posture, and in all 18 patients flow limited in the supine position, the tidal expiratory $\dot{V}-V$ loops on air and heliox_21_ were essentially superimposed, indicating that the choke point was located in the peripheral airways. In 4 flow-limited patients in the sitting position, heliox_21_ actually abolished flow-limitation, pointing at a central localization of the choke point. In these patients, flow-limitation actually involved the last fraction of the tidal volume (*V*_*T*_), so that no increase of inspiratory capacity was detected during heliox_21_ breathing. All the flow-limited patients remained flow-limited after salbutamol administration, nevertheless dynamic hyperinflation decreased as documented by the increase of inspiratory capacity, in line with what was previously reported [22]. As ventilation did not change after bronchodilator, the increase of inspiratory capacity was entirely due to higher maximal expiratory flows in the *V*_*T*_ range. In the non flow-limited patients at rest, neither heliox_21_ nor salbutamol caused inspiratory capacity to increase, simply because in these patients little or no dynamic hyperinflation is present at rest [36].

## 6. Heliox Breathing during Exercise in COPD Patients

 COPD patients are limited in their daily activity because of exercise intolerance due to dyspnea and/or leg fatigue. As the disease worsens, physical activities are progressively reduced, causing further deconditioning and worsening quality of life. Rehabilitation can potentially interrupt this vicious cycle. To be effective, rehabilitation should be performed at a sufficiently high level of exercise, and heliox has been regarded as a promising non pharmacological tool to improve exercise tolerance of COPD patients during rehabilitation programs.

 A number of different experimental approaches have been used to assess the effects of heliox_21_ breathing in exercising COPD patients, namely, (a) incremental work rate test on a cycle ergometer [37–40] or on a treadmill [41, 42], (b) constant work rate test on a cycle ergometer [30–35, 37, 43, 44], and (c) endurance shuttle walking test [45].

 In COPD patients cycling at increasing work rates, heliox_21_ increased maximal work rate in one study only [39] out of six [37–42], and ventilation at peak exercise in three studies [38–40] out of five [37–41]. At peak exercise, dyspnea [39, 40] and leg discomfort sensations [39] were not affected by heliox_21_.

 When COPD patients cycled to exhaustion at constant load, heliox_21_ increased exercise tolerance in five [30, 31, 33–35] out of six studies [30, 31, 33–36]. At isotime, ventilation was usually unaffected by heliox_21_ [30–33, 43], being, relative to air, increased in only two studies [35, 44] and decreased in only one [34]. In contrast, at peak exercise, ventilation was increased during heliox_21_ breathing [30, 31, 34, 35] except than in two studies [33, 37]. Dyspnea sensation was constantly decreased by heliox_21_ at isotime [30–35, 43, 44], while leg discomfort was decreased [33–35, 43, 44] or unchanged [30, 31]. At isotime, heliox_21_ was able to decrease exercise-induced dynamic hyperinflation in five studies [30, 31, 33, 34, 43] out of eight [30–35, 43, 44]. Of these eight trials, Vogiatzis et al. [44] did not observe any dynamic hyperinflation on air. In the COPD patients studied by Chiappa et al. [35], heliox_21_ markedly increased inspiratory capacity at rest (from 1.85 L in air to 2.17 L in heliox_21_). At isotime and peak exercise inspiratory capacity decreased relative to the rest value more during heliox_21_ breathing (−0.22 and −0.25 L, resp.) than during air breathing (−0.10 and −0.13 L, resp.).

A negative correlation between heliox-induced changes of dyspnea and inspiratory capacity at isotime has been found by Palange et al. [30], as expected according to the strict relation between dynamic hyperinflation and dyspnea. Eves et al. found that the decrease of dynamic hyperinflation with heliox_21_, together with the increase of peak expiratory flow and the reduction of total work of breathing, explained 99% of the variance associated with increased endurance time [31]. Similar results concerning the relation between dynamic hyperinflation and exercise tolerance have been obtained by other studies [34, 35].

 Heliox_21_ breathing increased markedly the endurance shuttle walking distance [45], to the same extent than 28% oxygen in nitrogen. In the same study, heliox_28_ provided further improvement relative to heliox_21_ or to 28% oxygen in nitrogen alone. The additive effects of helium and hyperoxia on exercise tolerance were later confirmed by Eves et al. [31]. In another research [46], heliox_30_ improved the 6-min walking distance more than 100% oxygen. A study in which training on heliox_40_ was compared with training on air was promising [47], showing that training on heliox_40_ increased exercise tolerance and quality of life more than training on air. A following study, however, did not confirm these results [48].

 Even if part of the contrasting results obtained can be related to differences in the experimental methodology [49, 50], most of the discrepancies probably depend on the heterogeneity of COPD patients. A potential confounding factor is the eventual presence of tidal expiratory flow-limitation [18], which has been investigated, using the negative expiratory pressure technique [51], only in one instance [32]. This study assessed, in 26 stable COPD patients, tidal expiratory flow-limitation, inspiratory capacity, breathing pattern, and dyspnea sensation during air and heliox_21_ breathing at rest and during exercise at 1/3 and 2/3 of the maximal work rate. On air, the patients who were flow-limited at rest remained flow-limited during exercise. In contrast, 4 and 7 of the patients who were not flow-limited at rest became flow-limited at 1/3 and 2/3 of maximal work rate, respectively. Dynamic hyperinflation was absent in the non flow-limited patients and developed only in the presence of flow-limitation. At rest, no difference was found between the breathing pattern of flow-limited and non flow-limited patients, while during exercise tidal volume increased more in non flow-limited patients. Heliox_21_ did not abolish flow-limitation, had no systematic effect on breathing pattern, and reduced dynamic hyperinflation in only 25% of the flow-limited patients. A positive correlation was found between the increase of end-expiratory lung volume on air and the reduction of dynamic hyperinflation induced by heliox_21_. This finding suggests that the heliox_21_ responders are those patients who during exercise increase their operational lung volume enough so that the choke point moves from peripheral to more central airways, where the maximal flows are determined by the wave-speed mechanism and are density-dependent.

 Dyspnea sensation was relieved by heliox_21_ in both flow-limited and non flow-limited patients, regardless of the presence or the absence of dynamic hyperinflation. In this connection, it should be underlined that dyspnea is not necessarily related to dynamic hyperinflation, in fact, in normal subjects, dyspnea may not change with heliox_21_ even if dynamic hyperinflation decreases [26], and, in COPD patients, dyspnea can decrease even in the absence of dynamic hyperinflation [32, 44]. The reduction of dyspnea documented by D'Angelo et al. [32] could thus be related to a decrease of the inspiratory work, which, depending on the extent of turbulence in the airways, can amount up to 50%–60% [6, 52].

## 7. Modelling Heliox Effects on the Respiratory System

 Because of the complex behaviour of the respiratory system especially in the presence of expiratory flow-limitation and the difficulty to directly assess the relevant variables in the human subject, mathematical models of the respiratory system have been developed and used to interpret the result of experimental research. Recently, a nonlinear dynamic mathematical model of the respiratory system, including both wave-speed and viscous mechanisms determining flow-limitation, was developed by Barbini et al. [53], on the basis of Weibel symmetrical morphometric description of the tracheobronchial tree [9] and on the mechanical characteristics of airway generations reported by Lambert [54]. This model has been used to simulate the response of the respiratory system to heliox_21_ in the presence of different obstructive conditions, all causing tidal expiratory flow-limitation [52]: (A) moderate to marked increase of the collapsibility of the peripheral airways (i.e., airways beyond the 7th generation); (B) marked increase of the collapsibility of peripheral airways with moderate involvement of the central ones (form the 4th to 7th generation); (C) markedly increased collapsibility of the central and peripheral airways; (D) markedly increased collapsibility of the central airways with moderate involvement of the peripheral ones. The effects of heliox_21_ have been evaluated in terms of inspiratory interrupter resistance  (*R*_int⁡_), intrinsic positive end-expiratory pressure (PEEPi), dynamic hyperinflation and expiratory flow-limitation.

 Heliox_21_ administration reduced *R*_int⁡_ in all cases except case A, where the viscous pressure loss was entirely due to laminar flow. The decrease of *R*_int⁡_ in case B, C, and D was considerable, amounting to 22% in case B and 27% in case C and D. Thus heliox_21_ should reduce the inspiratory work of breathing, accounting, at least in part, for the reduction of dyspnea sensation which has been reported in COPD patients especially during exercise [29].

 In no instance heliox_21_ abolished expiratory flow-limitation.

 PEEPi and dynamic hyperinflation decreased with heliox_21_ only trivially in case A (~1 and ~7%, resp.), where flow was limited by the viscous mechanism. Similar results were obtained for case B, even if the relative contribution of the viscous over the wave speed mechanism becomes relevant in the last part of the expiration only. In case C, the decrease of PEEPi and dynamic hyperinflation was modest (22 and 23%, resp.) because the contribution due to peripheral resistance to the total resistance of the upstream segment remained elevated. The fall of PEEPi and dynamic hyperinflation was remarkable (41 and 41%, resp.) in case D only, where flow limitation was dominated by the wave speed mechanism and the resistance of the peripheral airways was only slightly increased.

 Note that case A, B, and C can be regarded as three subsequent stages of chronic obstructive pulmonary disease, which initially involves the peripheral airways and then spreads to the whole tracheobronchial tree. Conversely, case D may represent severe asthma with mild involvement of peripheral airways or mild chronic obstructive pulmonary disease affecting mostly the central airways.

## 8. Conclusions

The administration of heliox, which is costly and cumbersome, to stable COPD patients at rest with moderate to severe disease is not warranted, because no beneficial effect in terms of breathing pattern or dynamic hyperinflation has been observed in most of the published trials. In contrast, heliox could be effective as non pharmacological tool to enhance the efficacy of rehabilitation programs, since its administration to COPD patients usually enhances their exercise tolerance, at least at constant work rate, and thus can be useful to increase the level of physical training. The conflicting results which have been obtained so far suggest that further research is needed in order to identify the COPD patients potentially able to benefit from this kind of rehabilitation programs.
